# Association between food insecurity and metabolic syndrome in North West of Iran: Azar Cohort study

**DOI:** 10.15171/jcvtr.2019.33

**Published:** 2019-08-22

**Authors:** Elnaz Faramarzi, Mohammadhossein Somi, Alireza Ostadrahimi, Saaed Dastgiri, Mousa Ghayour Nahand, Mohammad Asgari Jafarabadi, Sarvin Sanaie

**Affiliations:** ^1^Liver and Gastrointestinal Diseases Research Center, Tabriz University of Medical Sciences,Tabriz, Iran; ^2^Nutrition Research Center, Tabriz University of Medical Sciences, Tabriz, Iran; ^3^Social Determinants of Research Center, Tabriz University of Medical Sciences, Tabriz, Iran; ^4^Department of Statistics and Epidemiology, Faculty of Health, Tabriz University of Medical Sciences, Tabriz, Iran; ^5^Tuberculosis and Lung Disease Research Center, Tabriz University of Medical Sciences, Tabriz, Iran

**Keywords:** Food Security, Hunger, Metabolic Syndrome, Food Insecurity

## Abstract

***Introduction:*** Nowadays, prevalence of metabolic syndrome (MetS) is increasing in the world. There are inconsistence findings about the relationship between food insecurity and MetS. Therefore, the aim of this cross-sectional study was to determine the association between food insecurity and MetS in North West of Iran.

***Methods:*** The anthropometric measurements, food insecurity, dietary intake, blood pressure, fasting blood glucose (FBS), serum triglyceride and HDL levels of 151 subjects who had participated in Azar cohort study were evaluated. Food security was assessed by Household Food Security Scale (HFIAS) (six-item short questionnaire) and dietary intake (using 24- hour recall questionnaire) of participants. MetS was defined according to National Cholesterol Education Program’s Adult Treatment Panel III report (ATPIII ) criteria.

***Results:*** On the basis of HFIAS and energy, 7.3% and 11.9% of participants were food insecure and hunger, respectively. We observed no significant differences in mean body weight, BMI, waist circumference and FBS between food insecure and secure groups. Moreover, obesity (41.7% vs 30.2%) and MetS (45.5% vs 30%) were more prevalent in the food insecure group but the differences were not significant.

***Conclusion:*** The most percent of participants in food insecure were obese and had MetS. However, we could not find significant differences between food insecure and food secure groups. Therefore, for achieving more clear results, further studies with large sample size are needed.

## Introduction

In recent decade, people in developing countries intend to westernize leading to changes in their life style. The main effect of westernization is on food habit and physical activity of people in these countries.^1,2^ This type of changes in food habit is named nutritional transition^3^ which has many negative effects. Inadequate intake of micronutrients,^4^ increased incidence of metabolic syndrome (MetS) and chronic diseases are the important negative effects of nutritional transition.

Nowadays, MetS has become a major public health problem in the world. Its prevalence is increasing in developed and especially in developing countries.^5^ The prevalence of MetS varies between 16.3% and 33.4% in African and Asian countries.^6^ Moreover, Dalvand et al reported that prevalence of MetS was 38%^7^ based on the International Diabetes Federation (IDF) criteria in Iran. MetS increases the incidence of diabetes, cancer, cardiovascular diseases, etc.

On the other hand, it seems that rapid growth in economic status in upper–middle-income developing countries decreases food insecurity in these countries. The United States Department of Agriculture (USDA ) defines food security as “access to enough food by all people at all times for an active, healthy life.”^8^ It has been suggested that food insecurity increases the risk of obesity and MetS.^9^

Parker et al noted that marginal food insecurity and very low food insecurity increased risk of MetS by 1.80 and 1.65, respectively.^10^

The results of another study showed that there was a significant correlation between food insecurity and obesity among Mexican-American women.^11^

Albeit positive association between food insecurity, obesity and MetS have not been reported by other studies. In this regard, Shariff et al found that MetS components, over weight and obesity were more prevalent in those who were food secure than those with food insecurity.^12^

However, there are a few studies which have been examined the association of food insecurity and MetS. Moreover, in most studies, assessment of the food insecurity and chronic disease or MetS has been performed in low economic status region of the city or rural area. Therefore, we decided to determine the association between food insecurity and MetS in North West of Iran.

## Materials and Methods

### Setting

This study was carried out in Shabestar city which is located in North West of Iran. Shabestar is the capital city of Shabestar county, East Azerbaijan province, Iran. At the 2011 census, its population was 25,663, in 4824 families. It is located in proximity to Tabriz, the provincial capital, on the main Iranian-International railway line which connects Tehran and Tabriz to Turkey and Europe. Due to proximity to Lake Urmia, the city experiences mild weather with wet winters and summers are somewhat hot and dry during day time and cooler at night.

### Subject and sampling

In this study, we evaluated anthropometric measurements, food insecurity and dietary intake of 151 subjects who had participated in Azar cohort study. We have used convenience sampling method for recruiting study subjects. Azar cohort study is part of a large Persian cohort study (The Prospective Epidemiological Research Studies of the Iranian Adults)^13,14^ which has been launched in October 2014 and finished in January 2017. Azar cohort was established in Shabestar in East Azarbaijan province (North-west of Iran). Azar cohort has 3 phases which includes pilot, enrollment and follow-up phase. All participants signed a written informed consent.

All eligible individuals who aged35-70 years in Shabestar region were invited to participate in the study and those who signed the consent form were included in the Azar cohort study. Azar cohort study has been explained with more details in other published article.^15^ Demographic information of participants including age, gender, marital status, and education level were collected by data gathering form.

### Metabolic syndrome definition

MetS was defined according to the National Cholesterol Education Program’s Adult Treatment Panel III report (ATPIII) criteria.^16^ Subjects with three or more of the following conditions were considered as having the MetS: Waist circumference ≥102 cm in men and ≥88 cm in women; triglyceride (TG) ≥150 mg/dL (drug treatment for elevated triglycerides is an alternate indicator); High-density lipoprotein (HDL) cholesterol <40 mg/dl in men and <50 mg/dL in women; systolic blood pressure (SBP) ≥130 and/or diastolic blood pressure (DBP) ≥85 mm Hg (antihypertensive drug treatment in a patient with a history of hypertension is an alternate indicator); fasting glucose≥100 (drug treatment of elevated glucose is an alternate indicator).

### Biochemical factors

Blood samples were collected after an overnight fast of 12 hours. Fasting blood sugar (FBS), serum TG, HDL were determined by enzymatic method.

### Anthropometric measurements

According to standard protocols, mounted tape was used for measuring the height to the nearest 1 mm and Seca scale was used for recording weight to the nearest 0.1 kg. Body mass index was calculated by dividing weight (in kilogram) by the square of height (in meter). Waist circumference (WC) of subjects was measured according to NIH guidelines.^17^

### Blood pressure definition

The blood pressure was measured (by Riester sphygmomanometer, Germany) twice in each arm in the sitting position and according to Persian cohort protocol.^18^ Subjects with SBP ≥130 and DBP ≥85 or use of antihypertensive drug with a history of hypertension were considered as patients with hypertension.^19^

### Food security

Food security in this study was assessed by Household Food Security Scale (six-item short questionnaire) and dietary intake of participants.

Household Food Security Scale (six-item short questionnaire) is a valid scale for assessing food insecurity.^20^ If the subjects were responded to two or more of the six items, they were classified as food insecure.

Energy requirement of subjects were calculated by estimated energy expenditure prediction equation from Institute of Medicine, Food and Nutrition Board,^21^ and then compared with energy intakes of the subjects. Moreover, their protein, calcium, vitamin A and B2 intake were compared with dietary reference intakes (DRIs). Finally, energy intake ≥120% DRI was defined as over consumption; 90%-119% DRI as food secure; 80%-89% DRI as mild food insecure; and <80% DRI as sever food insecure (hunger).^22^

### Dietary intake

Dietary intake of participants was assessed by two days food record diary and 24-hour recall questionnaires. In food record diary and 24-hour recall method, subjects were asked to record all consumed foods and beverages in the previous 24 hours.^23^ Each food items was converted to g/d and then analyzed by Nutritionist IV. Nutritionist IV was used for determining the amount of energy, macro and micro nutrients intakes.

### Socioeconomic status (SES)

Socioeconomic status was defined based on job categories, education levels, and the family assets by using principal component analysis. SES was classified into very high, high, middle, low and very low, based on quintuple of obtained scores.^24^

### Statistical analysis

The data were analyzed using Statistical Package for the Social Sciences (SPSS, version 11.5, Chicago, IL). Descriptive statistics were obtained for all study variables and they were reported as mean ± SD and also number (percent) where applicable. The normality of data was assessed using Kolmogorov-Smirnov test. Independent *t* test, chi-square and one-way ANOVA were used to analyze data. A *P* value less than 0.05 was statistically considered significant. Confounding effects of age, gender, SES and energy intake were adjusted by Univariate test, ordinal and logistic regression.

## Results

Baseline characteristics of participants are presented in Table 1. The mean age of subjects was 52.88±9.08 years. Overweight was observed in 49.4% and 47% of women and men, respectively. On the basis of HFIAS, 11 (7.3%) participants was food insecure. As a point of energy intake, 8 (12.1%) of men and 10 (11.8%) of women were sever food insecure (hunger). As compared with DRI, protein, calcium, vitamin A and B2 intake of 37 (24.5%), 125 (82.8%), 77 (51%) and 55 (36.4%) of participants were lower than 80% of DRI (Table 2).

**Table 1:** Baseline characteristics of participants (n=151)

**Variable**	**Males (n=66)**	**Females (n=85)**	**Total (n=151)**	**P value**
Age (y)	54.08±8.93	51.95±9.15	52.88±9.08	0.15^*^
Weight (kg)	78.90±13.82	73.5±13.74	75.86±13.99	0.01^*^
Height (cm)	169.38±7.97	158.59±7.38	163.31±9.32	<0.001^*^
Waist (cm)	93.92±11.05	89.88±11.27	91.46±11.32	0.02^*^
BMI (Kg/m^2^)	27.49±4.57	29.22±5.14	28.46±4.96	0.03^*^
BMI (kg/m^2^) classification				
Underweight, No. (%)	1 (1.5)	-	1 (0.7)	0.09**
Normal (18.5-24.9)	18 (27.3)	12 (14.1)	30 (19.9)	
Overweight (25-29.9)	31 (47)	42 (49.4)	73 (48.30)	
Obese (≥30)	16 (24.2)	31 (36.5)	47 (31.10)	
Education level, No. (%)				
Illiterate	3 (4.5)	7 (8.2)	10 (6.60)	0.15**
≤High school/diploma	50 (75.8)	69 (81.2)	119 (78.8)	
≥College degree	13 (19.6)	9 (10.6)	22 (14.70)	
Socioeconomic status, No. (%)				
Very high	17 (25.8)	17 (21.5)	34 (23.5)	0.72**
High	16 (24.2)	20 (25.3)	36 (24.8)	
Middle	14 (21.2)	13 (36.5)	27 (18.6)	
Low	5 (7.6)	11 (13.9)	16 (11.0)	
Very low	14 (21.2)	18 (22.8)	32 (21.2)	
Marital status, No. (%)				
Single	1 (1.5)	3 (3.5)	4 (2.60)	0.08**
Married	64 (97)	74 (87.1)	138 (91.40)	
Widowed	1 (1.5)	8 (9.4)	9 (6)	

N=number; *P value: independent *t* test; **P value: chi-square.

**Table 2:** Comparison of protein and key micronutrients intake of participants with dietary intake allowance (DRI)

	**Men (n=66)**		**Women (n=85)**		**Total (n=151)**	
	**<80% DRI**	**≥80% DRI**	**<80% DRI**	**≥80% DRI**	**<80% DRI**	**≥80% DRI**
	**No. (%)**	**No. (%)**	**No. (%)**	**No. (%)**	**No. (%)**	**No. (%)**
Protein	9 (13.6)	57 (86.4)	28 (32.9)	57 (67.1)	37 (24.5)	114 (75.5)
Vitamin A	35 (53)	31 (47)	42 (49.4)	43 (50.6)	77 (51)	74 (49)
Riboflavin (B2)	18 (27.3)	48 (72.7)	37 (43.5)	48 (56.5)	55 (36.4)	96 (63.5)
Calcium	47 (71.2)	19 (28.8)	78 (91.8)	7 (8.2)	125 (82.8)	26 (17.2)

As shown in Table 3, after adjusting the energy intake, the mean protein, calcium, vitamin A and riboflavin intake of hunger group were significantly lower than food secure group (*P*<0.05). Moreover, energy intake of food insecure (on the basis of HFIAS) group was higher than secure group.

**Table 3:** The mean energy, protein, calcium, vitamin A and B2 on the basis of different food security classification

	**HFIAS**		***P*** value* unadjusted	***P*** value** **adjusted**	**Energy intake (kcal/d)**			***P*** value* unadjusted	***P*** value** **adjusted**
	**Food secure** ** (n=140)**	**Food insecure** ** (n=11)**			**Food secure** ^ 1 ^ ** (n=100)**	**Mild insecure** ^ 2 ^ ** (n=33)**	**Sever insecure (hunger)** ^ 3 ^ ** (n=18)**		
	**Mean±SD**	**Mean±SD**			**Mean±SD**	**Mean±SD**	**Mean±SD**		
Energy (kcal/d)	1833±612	1970.2±656	0.47	-	2016.38±568^§^	1614.9±683.4	1443.9±484	<0.001	-
Protein (g/d)	64.±23.25	58.79±27.7	0.54	0.33	63.8±28.77^§§^	53.6±23.71	48.3±21.2	0.01	0.49
Calcium (mg/d)	604.43±178.21	545.3±266.7	0.47	0.99	576.31±274.2	890.5±124	515±245	0.18	<0.001
Vitamin A (mcg/d)	1119.74±1300	1090±1024	0.87	0.63	1232.3±1412.4	608.2±23.6	928.9±960	0.29	0.02
B2 (mg/d)	1.43±3.04	1.29±0.52	0.94	0.67	1.16±3.6	0.91±0.02	0.99±0.35	0.47	0.03

*P value: independent test; **P adjusted for energy intake by Univariate test; ^§^ Post hoc *P* value (0.001): significantly different from mild insecure group (0.001); ^§§^ Significantly different from mild insecure group (0.005). ^1^Food secure; energy intake 90-119% DRI; ^2^Mild insecure; energy intake 80%-89% DRI; ^3^ Sever food insecure (hunger); energy intake <80% DRI.

Distribution of body mass index (BMI) and MetS has been indicated in Table 4. We observed the mean body weight, BMI, waist circumference and FBS of food insecure group were insignificantly higher than food secure group. Moreover, obesity (41.7% vs. 30.2%) and MetS (45.5% vs. 30%) were more prevalent in the food insecure group (41.7% vs. 30.2%).

**Table 4:** Distribution of BMI and metabolic syndrome on the basis of food insecurity classification

	**HFIAS**		***P*** **unadjusted**	***P*** *** **adjusted**	**Hunger**			***P*** **unadjusted**	***P*** *** **adjusted**
	**Food secure (n=140)**	**Food insecure (n=11)**			**Food secure** ^1^ ** (n=100)**	**Mild insecure** ^2^ ** (n=33)**	**Sever insecure** ^3^ ** (n=18)**		
Weight	75.6±13.5	78.4±19.4	0.55^*^	0.51	71.2±8.6	70.9±13.5	76.6±14.2	0.4^*^	0.15
BMI (kg/m^2^)	28.4±4.8	29.5±6.1	0.6^*^	0.39	26.4±2.80	27.1±4.6	28.7±5.0	0.36^*^	0.17
WC (cm)	91.32±11.1	95.6±13.6	0.23^*^	0.15	87.8±9.6	88.4±11.0	92.1±11.4	0.45^*^	0.23
FBS (mg/dL)	105.9±48.4	110.3±36.4	0.75^*^	0.2	111.6±58.5	97.5±18.9	107.0±48.8	0.66^*^	0.38
Triglycrides (mg/dL)	158.2±78.5	146.0±46.1	0.6^*^	0.3	152.1±54.9	127.2±50.3	160. 79.3	0.51^*^	0.63
HDL (mg/dL)	48.9±11.2	45.6±9.8	0.23^*^	0.3	52.3±11.3	48.8±6.5	48.4±11.4	0.22^*^	0.77
Blood pressure									
Systolic (mm Hg)	108.1±15.0	101.0±10.2	0.24^*^	0.22	105.0±11.4	105.2±19.2	107.9±14.6	0.7^*^	0.19
Diastolic (mm Hg)	69.2±8.9	66.1±6.9	0.24^*^	0.51	69.7±10.5	66.1±7.2	69.2±8.7	0.5^*^	0.36
BMI classificatin			0.80^**^	0.33				0.30^**^	0.11
Under weight (<18.5)	1 (0.7)	-			1 (1)	-			
Normal (18.5-24.9)	28 (20.1)	2 (18.8)			17 (17)	10 (30.3)	3 (16.7)		
Over weight (25-29.9)	68 (48.9)	4 (36.3)			55 (55)	12 (36.4)	6 (33.3)		
Obese (≥30)	42 (30.2)	5 (41.7)			27 (27)	11 (33.3)	9 (50)		
Metabolic syndrome	42 (30)	5 (45.5)	0.51^**^	0.14	28 (28)	3 (25)	43 (33.1)	0.33	0.04

Abbreviations: BMI, body mass index; WC, Waist circumference; FBS, Fasting blood sugar. *P value: independent test; **P value: chi-square test; *** *P* adjusted for age, gender, Socioeconomic status; quantitative variables were adjusted for age, gender and SES by Univariate test; nominal and ordinal variables were adjusted for age,gender and SES by ordinal and binary regressions respectively. ^1^Food secure; energy intake 90-119% DRI; ^2^Mild insecure; energy intake 80-89% DRI; ^3^ Sever food insecure (hunger); energy intake <80% DRI.

Defining food insecurity on the basis of energy intake as hunger showed that the anthropometric measures, blood pressure and serum TG were higher in this group than the other groups (food secure and mild insecure) (Table 4). In addition, a great number of subjects in the severe food insecure group were obese (50%). We observed that 33.1% of food insecure group had MetS which was significantly higher than food secure group after adjusting for age, gender and SES (*P*=0.04).Computing percent difference by different assessment scales indicated that the difference between food insecure and food secure in obesity and MetS are more than 10% in all classification of food security.

## Discussion

Nowadays, despite the rapid growth in economic statuses in developing countries, food security issue remains as a major concern.

The results of HFIAS indicated that only 7.3% of participants were sever food insecure.

In line with the present result, Salarkia and other reported by 7.5% of evaluated household by HFIAS were sever food insecure.^25^ In contrast to our findings, the results of systematic review about the prevalence of food insecurity in Iran, showed food insecurity is high and by 49% of household were food insecure.^26^ Moreover, it has been reported that by 85.2% of Malaysian palm plantation household were food insecure.^27^

The observed discrepancy in the findings of studies may be due to different scales which were used to assess food insecurity, different sample size, rural vs. urban area and various economic statuses.

Another finding of this study showed that by 11.9% of subjects were hunger (<80% of energy requirements). The findings of two large studies in Iran showed that by 10% and 12.5% of household consumed <80% of energy requirements in 1993 and 2004, respectively.^28,29^

Ghassemi et al noted that by 20% and 50% of people have problem in providing their energy and micronutrients requirements,respectively.^30^

The comparison of our results with their study indicated that the prevalence of hunger decreased. It seems that the differences can be attributed to rapid growth in economic status and nutrition transition which take place in recent decades in Iran.^30^

These changes have its advantages and its disadvantages. Advantages are decreasing food insecurity in country; on the other hand, it results in inadequate intake of key micronutrients.

Interestingly, calcium intake of 71.2% of men and 91.8% of women were <80% DRI. Moreover, inadequate intake (<80% DRI) of vitamin A, B2 and protein was observed in 51%, 36.4% and 24.5% of subjects, respectively.

Similar to our results, Arsenault et al in a study on adequacy of micronutrient intakes by women in rural Bangladesh found that only <1% of women consumed adequate amount of calcium.

This finding was in line with the results of the study which evaluated inadequate intake of micronutrients in south Asia region and indicated that among seven assessed micronutrients, inadequate intake of calcium was more evident in this region.^4^ In the mentioned study, Iran has the highest intake for vitamin A, folate and B2 among seven countries. For example, prevalence of vitamin A inadequacy in comparison to EAR (estimated average requirement) was by 78% for Bangladesh and by 1% for Iran^4^. These findings were in contrast with our results which inadequate intake of vitamin A (<80% DRI) was by 51%. The differences can be ascribed to references which are used to compare intake of micronutrients with them (EAR vs. DRI).

Additionally, findings of national surveys about changes in food basket in urban area indicated that consumption of meat, egg, dairy products, fruit and vegetables remarkably decreased during 1985 to 1995. This reduction which was more evident for meat (from 138 to 65 g/d), fruit and vegetables (from 755 to 432 g/d),^31^ resulted in inadequate intake of protein, vitamin A and B2.

We thought that these changes were associated with nutrition transition in our country. In support of this idea, previous studies have documented that changes in dietary pattern and life style took place in most countries (Asia, Latin America, Northern Africa, the Middle East and the urban areas of sub-Saharan Africa) in last decades.^32-34^ Shifting in dietary pattern leads to overconsumption of high-caloric and low-nutrients foods. Therefore, key micronutrients deficiency is expected to happen in this population. In this regard, our findings showed that energy and protein intakes were higher but micronutrients intake was lower in food insecure group than food secure group.

In this cross-sectional study, the mean body weight, BMI and waist circumferences were high in food insecure and hunger groups. These findings are consistence with Bawadi et alr^31^ who found BMI of patients were higher in food insecure group than food secure group (34.9 vs. 32.6 kg/m^2^). Moreover, Cheung and other observed that food insecurity resulted in increasing BMI during 3.2 year follow up (0.15 kg/m^2^ per year more than controls).^35^ Also, findings of another large study indicated that food insecurity was significantly correlated with overweight and obesity among Mexican-American women.^11^ However, we could not find significant differences between food insecure and food secure group. It may be due to small sample size.

A great number of participants had MetS in food insecure and hunger groups. Our results are in line with the previous studies which have been conducted about the correlation of food insecurity and MetS. These studies reported that there was a positive association between food insecurity and MetS.^10,36-40^

In addition, it has been reported that food insecurity is associated with type 2 diabetes and hypertension^40-42^; results of the present study indicated that the mean FBS levels of food insecure group were higher than food secure group.

Several hypotheses are involved in increased risk of MetS in food insecure population. They overcompensate when food is available; their basal metabolic rate is decreased in response to food shortage; and they consume inexpensive food which are energy-dense but low-nutrient.^43-45^ As a result, all mentioned factors lead to increase the risk of overweight, obesity, MetS and chronic disease.

The strength of this study was that food security has been assessed by two scales including HFIAS questionnaire and 24-hour recall method. Therefore, we can determine prevalence of food insecurity and hunger. Small sample size was limitation of the present study. Thus, because of small sample sizes among some subgroups, we were unable to achieve the power which was necessary to detect differences (household food insecurity as determinants).

Other limitation of the present study was that physical activity of subjects which was not evaluated with more details.

## Conclusion

On the basis of these findings, inadequate intake of key micronutrients especially calcium is the major problem in these people. On the other hand, more percent of participants in food in secure and hunger were obese and had MetS. However, we could not find significant differences between food insecure and food secure group. It may be due to small sample size. Therefore, for achieving more clear results, further studies are needed with large sample size.

Despite the increasing prevalence of MetS in the world, its etiology is not still understood fully. Various causes have been proposed in its etiology; food insecurity may be one of the causes as shown in our study. So, prevention of nutrition transition and food insecurity can be one of the ways to decrease incidence of MetS. To achieve this goal, it can be suggested to increase the knowledge of population about their food security and nutrition literacy.

## Ethical approval

This cross-sectional study was approved by Ethics Committee of Tabriz University of medical sciences (tbzmed.rec.1393.205).

## Competing interests

The authors declared no potential conflicts of interest with respect to the research, authorship, and/or publication of this article.

## Funding

The authors disclosed receipt of the following financial support for the research, authorship, and/or publication of this article: the Social Determinants of Research Center, Tabriz University of Medical Sciences.

## Acknowledgements

The authors also are deeply indebted to all subjects who participated in this study. We appreciate the contribution by investigators and staff of the Azar Cohort Study. We thank the close collaboration of the Shabestar Health center.
